# Interplay of Thermo-Optic and Reorientational Responses in Nematicon Generation

**DOI:** 10.3390/ma11101837

**Published:** 2018-09-27

**Authors:** Urszula A. Laudyn, Armando Piccardi, Michal Kwasny, Bartlomiej Klus, Miroslaw A. Karpierz, Gaetano Assanto

**Affiliations:** 1 Faculty of Physics, Warsaw University of Technology, PL-00662 Warsaw, Poland; ulaudyn@if.pw.edu.pl (U.A.L.); mkwasny@if.pw.edu.pl (M.K.); bartklus@if.pw.edu.pl (B.K.); karpierz@if.pw.edu.pl (M.A.K.); 2 NooEL—Nonlinear Optics and OptoElectronics Lab, University Roma Tre, I-00146 Rome, Italy; assanto@uniroma3.it

**Keywords:** nematic liquid crystals, reorientational response, thermo-optic effect, optical solitons

## Abstract

Employing several nematic liquid crystal mixtures, we investigate how the thermo-optic response of nonlinear birefringent soft-matter affects the propagation of light beams and the features of self-induced waveguides. We address the formation of optical spatial solitons and the control of their trajectories versus temperature, comparing the measurements with the expectations based on a simplified model, showing an excellent agreement. Moreover, in a guest–host mixture with an absorbing dye dopant, we study the competition between reorientational and thermal nonlinearities, demonstrating that the two processes can be adjusted independently in order to tune the soliton properties, i.e., trajectory and confinement strength. Our results are an important contribution to better comprehend the role played by material properties on linear and nonlinear beam propagation, as well as their exploitation for signal processing and addressing.

## 1. Introduction

Liquid crystals are states of matter where some properties of liquids and solids can coexist, resulting in different phases depending on the strength of the intermolecular forces. The nematic mesophase features a finite degree of orientational order and lack of positional order of its anisotropic elongated molecules. Otherwise stated, they are randomly arranged in space, but their long (main) axes are oriented -on average- in a specific direction [1]. In the formalism of continuum theory, the nematic phase is described through the molecular distribution by introducing a unity vector, the so called molecular director **n**, and an order parameter defining the average direction and the variance of the molecular orientation, respectively. Macroscopically, nematic liquid crystals (NLC) exhibit anisotropic optical properties: when prepared on properly treated substrates, the order extends to long-range and they become uniaxial media with refractive indices $n_{⊥}$ and $n_{\left|\right|}$ for electric fields perpendicular and parallel to the molecular director **n**, respectively, with the latter taking the role of the optic axis [2].

When subjected to electric fields at either low or optical frequencies, the formation of dipoles tends to reorient the NLC molecules towards the electric field, altering the director distribution and, in turn, the dielectric tensor. In the case of bell-shaped light beams with finite size and in extraordinary-wave polarization (electric field vector coplanar with both the optic axis **n** and the wave-vector **k**) the transverse distribution of refractive index resulting from reorientation is focusing or even confining [3], with the beam power determining the magnitude of the nonlinear perturbation. When reorientational self-focusing balances linear diffraction, the beam can propagate without energy spreading and maintain an invariant profile: a spatial optical soliton or nematicon is generated [4]. Due to the elastic intermolecular forces, the refractive index perturbation extends well beyond the beam size, i.e., NLC are strongly nonlocal; hence, two-dimensional solitons are stable as the catastrophic collapse typical of Kerr nonlinear materials is avoided [5,6]. The large reorientational response and the high nonlocality of NLC favor the formation of 2D+1 spatial solitons with continuous-wave bell-shaped beams at milliwatt powers and waists of a few micrometers [7], even in the limit of spatial incoherence [8,9,10]. Owing to the guiding properties of the light-induced index channels and the NLC sensitivity to stimuli, nematicons have been employed as waveguides for co-polarized signals of different wavelengths, to be switched and steered by way of electric or magnetic fields [11,12,13,14,15,16], extra self-confined beams or refractive index perturbations [17,18,19,20,21], even self-induced changes in director distribution [22,23,24]. In this framework, nematicons are candidates for novel generations of electro-optic and all-optical devices based on or controlled by self-induced waveguides [25,26,27,28], including cavity-less lasers [29,30].

Reorientation is not the only nonlinear optical mechanism available in NLC. Among others, temperature variations affect the intermolecular links [31], acting on the order parameter [32] and thus varying both the mechanical and the optical responses through the elastic properties and the effective birefringence [33].

The typical NLC thermo-optic response is of sign opposite to the reorientation, i.e., it is defocusing for extraordinary waves (as well as focusing for ordinary waves), and can be employed in various ways [34,35,36] as it is nonlocal, as well [37,38,39,40]. Changes in the environmental temperature are able to tune both linear and nonlinear optical properties of NLC [41], while local heating due to absorbed light beams can yield self-focusing/defocusing and contribute to self-confinement [42].

These two main nonlinear contributions, namely reorientational and thermal, have been employed together in both synergistic and competing configurations for the generation and the control of spatial optical solitons in NLC [43,44].

In this work we report our results in several NLC mixtures exhibiting various optical birefringences as well as elastic properties, addressing the role of temperature in reorientational self-confined beam propagation: we show that temperature can affect the formation, trajectory and degree of confinement of nematicons; moreover, we use beam-induced thermo-optic effects in synergy with reorientation to control on the overall nonlinear response and so achieve a further degree of control.

## 2. Temperature Effects on Beam Propagation

**Figure 1:**
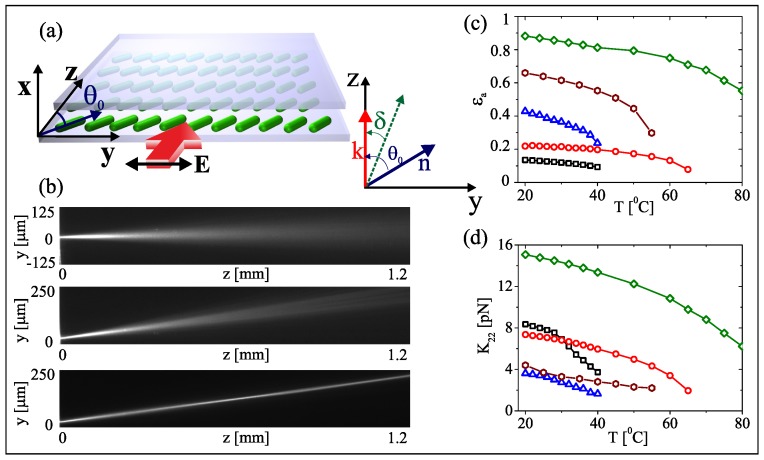
(**a**) NLC sample geometry. (**b**) Typical linear and nonlinear propagation of light beams. Top: ordinary-wave diffracting beam. Center: extraordinary-wave beam at low power, undergoing diffraction. Bottom: extraordinary-wave beam at high power, undergoing self-confinement. Measured (**c**) dielectric and (**d**) elastic constant $K_{22}$ versus temperature in five NLC mixtures: 1110 (black squares), 903 (red circles), 6CHBT (blue triangles), E7 (brown hexagons), 2007 (green diamonds).

In this section we consider an NLC sample of thickness *h* with director **n** uniformly oriented in the plane yz at an angle $\theta_{0}$ with the *z* axis, as sketched in Figure 1a. The optical excitation is a Gaussian beam launched with k along *z*. When polarized as an ordinary wave, the beam propagates along *z* and diffracts as in isotropic media, with refractive index $n_{o}=n_{⊥}$, as shown in the top panel of Figure 1b; conversely, the beam in the extraordinary-wave polarization propagates in a $\theta_{0}$-dependent refractive index $n_{e}={\left(\cos^{2}\theta_{0}/n_{⊥}^{2}+\sin^{2}\theta_{0}/n_{\left|\right|}^{2}\right)}^{-1/2}$, with energy flux (Poynting vector) angularly displaced by the walk-off $\delta \left(\theta_{0}\right)=\frac{1}{n_{e}}\frac{\partial n_{e}}{\partial \theta_{0}}$ from the wave vector k.

Both ordinary and extraordinary wave refractive indices (and therefore the walk-off) depend on temperature according to:

(1) $$\begin{array}{c} n_{\left|\right|}\left(T\right)\approx C_{1}-C_{2}T+\frac{2}{3}{\left(\Delta n\right)}_{0}\left(1-\frac{T}{T_{NI}}\right) \end{array}$$

(2) $$\begin{array}{c} n_{⊥}\left(T\right)\approx C_{1}-C_{2}T-\frac{1}{3}{\left(\Delta n\right)}_{0}\left(1-\frac{T}{T_{NI}}\right) \end{array}$$

where $C_{1},C_{2}$, the transition temperature $T_{NI}$ and the initial birefringence ${\left(\Delta n\right)}_{0}$ are material-dependent empirical parameters [45].

Thus, the propagation of the extraordinary-wave beam can be described by a nonlinear Schrödinger-like equation cast as [46]:

(3) $$\begin{array}{c} 2ik_{0}n_{⊥}\left(T\right)\left(\frac{\partial A}{\partial z}+\tan\delta \left(T\right)\frac{\partial A}{\partial y}\right)+\frac{\partial^{2}A}{\partial x^{2}}+D_{y}\frac{\partial^{2}A}{\partial y^{2}}+k_{0}^{2}\Delta n_{e}^{2}\left(\theta ,T\right)A=0 \end{array}$$

where $k_{0}$ is the vacuum wave-number, *A* is the amplitude of the slowly varying envelope of the magnetic field associated to the light beam and $D_{y}$ the diffraction coefficient across *y*. Equation (3) includes the walking-off Poynting vector and the nonlinear index change $\Delta n_{e}^{2}\left(\theta \right)=n_{e}^{2}\left(\theta \right)-n_{e}^{2}\left(\theta_{0}\right)$, both of which are temperature dependent.

In particular, $\Delta n_{e}^{2}$ depends on the director distribution, which in turn results from the balance between the light-induced torque (nonlinear response) and the restoring (elastic) molecular forces, as modeled by the reorientation equation [46]:

(4) $$\nabla^{2}\psi +\kappa^{2}\frac{\epsilon_{0}\epsilon_{a}\left(T\right)}{4K\left(T\right)}\sin\left[2\left(\theta_{0}+\psi -\delta \left(\theta_{0},T\right)\right)\right]{\left|A\right|}^{2}=0$$

where the optic-axis distribution is given by $\theta =\theta_{0}+\psi$, i.e., the superposition of the orientation at rest and the nonlinear reorientation ψ due to the beam. In Equation (4), $\kappa =Z_{0}/n_{e}\left(\theta_{0}\right)\cos\delta \left(T\right)$, with $Z_{0}$ and $\epsilon_{0}$ the vacuum impedance and dieletric constant, respectively, $\epsilon_{a}=n_{\left|\right|}^{2}-n_{⊥}^{2}$ the optical anisotropy, K(T) the Frank constant (in the scalar limit with equal coefficients for bend, twist and splay deformations [1]) quantifying the temperature-dependent strength of the intermolecular links. Equation (4) describes the material response to an optical excitation: at low power, reorientation is negligible and the extraordinary-wave beam propagates in the linear regime, spreading according to the extraordinary refractive index, as shown in the central panel of Figure 1b; when the power is high enough to cause reorientation, the nonlinear refractive index change (Equation (3)) can enable self-confinement and the generation of a spatial soliton, as visible in the bottom panel of Figure 1b. In Equations (3)–(4) we underline the temperature-dependent opto-mechanical parameters; in Figure 1c,d we plot $\epsilon_{a}$ and *K* measured in several NLC mixtures as a function of temperature.

Qualitatively, the heat damped in the medium through absorption increases the molecular oscillations and reduces the elastic forces, with a resulting larger reorientation for a given beam. This causes the beam trajectory to vary through changes in walk-off, the latter conveniently expressed as:

(5) $$\delta \left(T\right)=\arctan\left[\frac{\epsilon_{a}\left(T\right)\sin2\theta \left(T\right)}{\epsilon_{a}\left(T\right)+2n_{⊥}{}^{2}\left(T\right)+\epsilon_{a}\left(T\right)\cos2\theta \left(T\right)}\right]$$

Otherwise stated, since Equation (4) is temperature-dependent, so it is the electro-magnetic torque required to produce a finite director rotation. Considering an equivalent nonlinear coefficient defined as [46]

(6) $$n_{2}\left(T\right)=2\frac{\epsilon_{0}\epsilon_{a}\left(T\right)}{K(T)}\sin\left[2\left(\theta_{0}-\delta \left(T\right)\right)\right]n_{e}^{2}\left(\theta_{0},T\right)\tan\delta \left(T\right),$$

the nonlinearity, hence the degree of confinement of the solitary beam, is also temperature-dependent.

In dye-doped NLC where an absorbing guest is added to the NLC host [24,36,47], one has to account for the heating self-provided by the beam and model also the thermo-optic response. In this case a Poisson equation can be expressed as

(7) $$\nabla^{2}T=-\frac{\alpha^{\left(j\right)}n^{\left(j\right)}}{2\kappa Z_{0}}{\left|A\right|}^{2}\;j=\mathrm{ord}.,\mathrm{extr}.,$$

where α and *n* are the polarization dependent (superscript *j*) absorption coefficient and the refractive index, respectively, in order to address the thermal nonlinear response dependent on beam intensity, profile and polarization. Equations (3), (4) and (7) describe the nonlinear propagation of a light beam subject to competing nonlinearities stemming from reorientational and thermal responses, which modify the director and temperature distributions, respectively. Both affect the refractive index, and Equations (5) and (6) allow to quantify some macroscopic figures.

In the following we present experimental results on five NLC mixtures, identified as 1110 [48], 903 [48], 6CHBT [49,50], E7 and 2007, comparing expected and observed behaviors of propagating beams. These NLC mixtures, all synthesized at the Military University of Technology in Warsaw (Poland), feature different elastic constants, birefringence, refractive indices and cross-over temperatures.

In our experimental configuration, since the main texture deformation is a molecular twist, it suffices to consider the $K_{22}$ Frank constant. Its detailed measurements were carried out by us and the corresponding results are plotted versus temperature in Figure 1c. By increasing the sample temperature up to $60\;{}^{\circ }$C we observed quite appreciable decreases of birefringence and elastic constant, affecting beam propagation in nontrivial manners.

**Figure 2:**
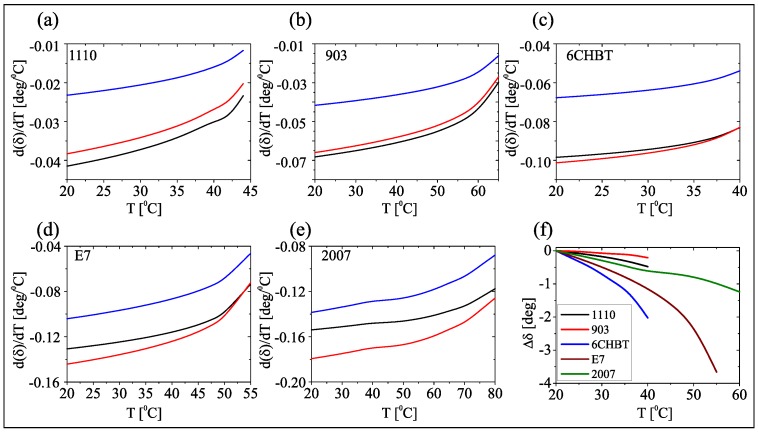
(**a**–**e**) Walk-off sensitivity to temperature for various $\theta_{0}$: black line $\theta_{0}=75^{\circ }$, red line $\theta_{0}=60^{\circ }$, blue line $\theta_{0}=45^{\circ }$ for the NLC mixtures as labelled and (**f**) walk-off variation versus temperature with respect to its reference value, in the five NLC mixtures: 1110 (black), 903 (red), 6CHBT (blue), E7 (brown), 2007 (green).

As it can be inferred from Equation (5), the initial angle $\theta_{0}$ determines the sensitivity of walk-off to temperature. As plotted in Figure 2, in low-birefringence NLC (1110 and 903) the choice of $\theta_{0}$ is not relevant, as walk-off changes are negligible; in high-birefringence mixtures the dependence is stronger (particularlyt at low temperatures) and $\theta_{0}=60^{\circ }$ maximizes the walk-off sensitivity. Consistently, for the experiments the NLC samples were prepared with **n** at $\theta_{0}=60^{\circ }$ with respect to the input beam wave-vector (k parallel to *z*).

In the next sections we show our results on temperature-dependent beam trajectory and confinement, addressing the interplay of reorientation and temperature. We first investigate the propagation of a single beam in various NLC mixtures as the temperature is changed, then we focus on nematicon propagation and its control in the case of competing nonlinearities in the presence of two input beams at different wavelengths in a dye-doped NLC.

## 3. Temperature-Dependent Nematicon Propagation

**Figure 3:**
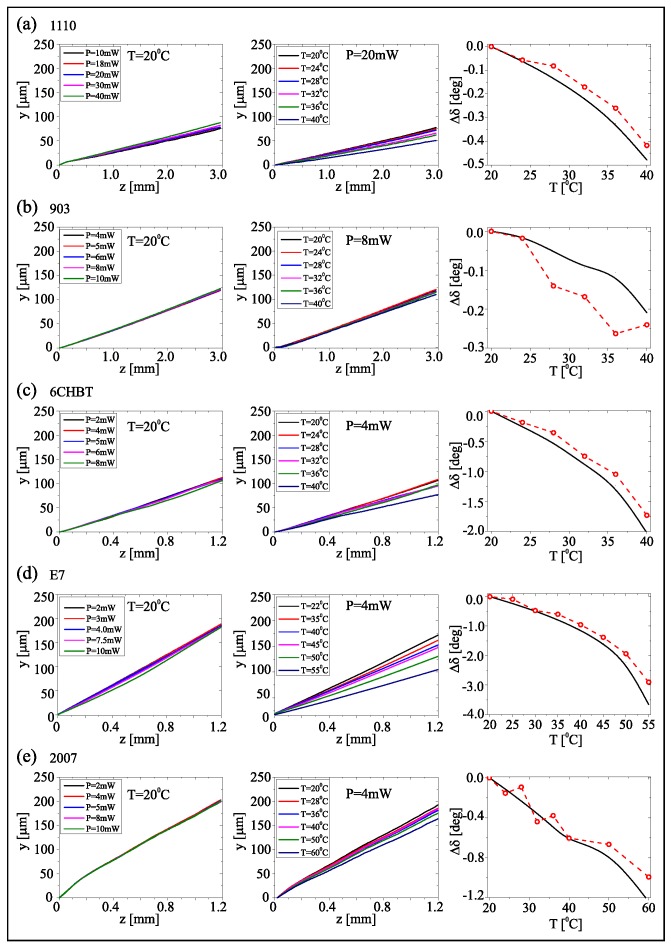
(**Left**): beam trajectory vs. power. (**Center**): beam trajectory vs. temperature. (**Right**): comparison of calculated and measured walk-off changes with respect to its initial value. Rows (**a**–**e**) refer to the five mixtures 1110 (**a**), 903 (**b**), 6CHBT (**c**), E7 (**d**), and 2007 (**e**), respectively.

According to Equations (5) and (6), walk-off and confinement of propagating beams depend on material properties. To experimentally ascertain the corresponding trends, we launched a $TEM_{00}$ mode from a near-infrared laser at λ=1.064 μm in NLC samples, as sketched in Figure 1a. Upon varying input power and temperature, we monitored the beam evolution in the plane *yz* in order to evaluate the material properties. First we measured the walk-off of extraordinary-wave beams as a function of temperature. For each of the mixtures we employed a high enough power to generate a nematicon, maximizing beam visibility versus z in the absence of diffraction. Such measurements are summarized in Figure 3.

Within the range of powers used, we verified that the beam trajectories were power-independent with walk-off defined by the linear properties of the material, as visible on the left of Figure 3. Upon changes in temperature, the trajectory varied (center column in Figure 3) according to the acquired values of refractive indices and birefringence. Except for the mixture 903, in which the extremely low walk-off (≈$0.5^{\circ }$) could not be appreciated, all measurements showed the expected decrease, as seen on the right of Figure 3 where data are compared with predictions from Equation (5).

**Figure 4:**
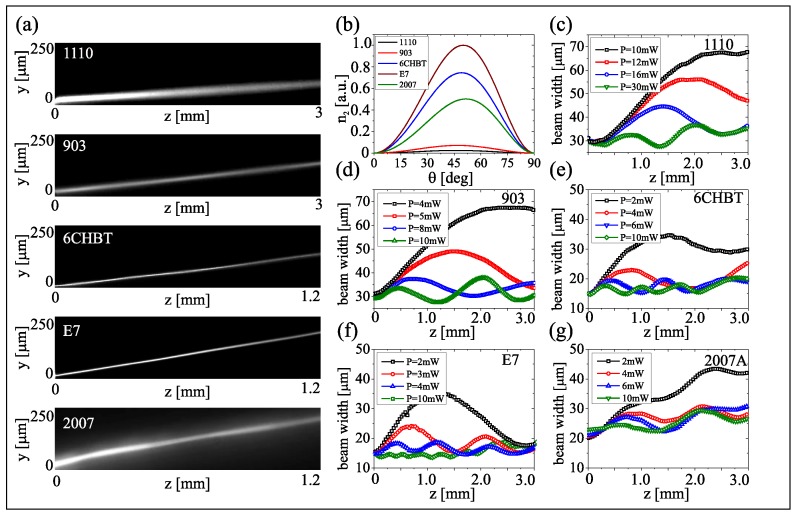
(**a**) Acquired images of P=10 mW nematicon propagation in the five NLC mixtures, as labelled. (**b**) Calculated nonlinearity versus $\theta_{0}$ for each material. (**c**–**g**) Beam-width evolution versus propagation for various input powers (see legends), and for the five mixtures, as labelled. As power increases, the breathing period decreases. The relative values are coherent with the graphs in panel (**b**): a larger nonlinear figure is associated to a shorter period.

**Figure 5:**
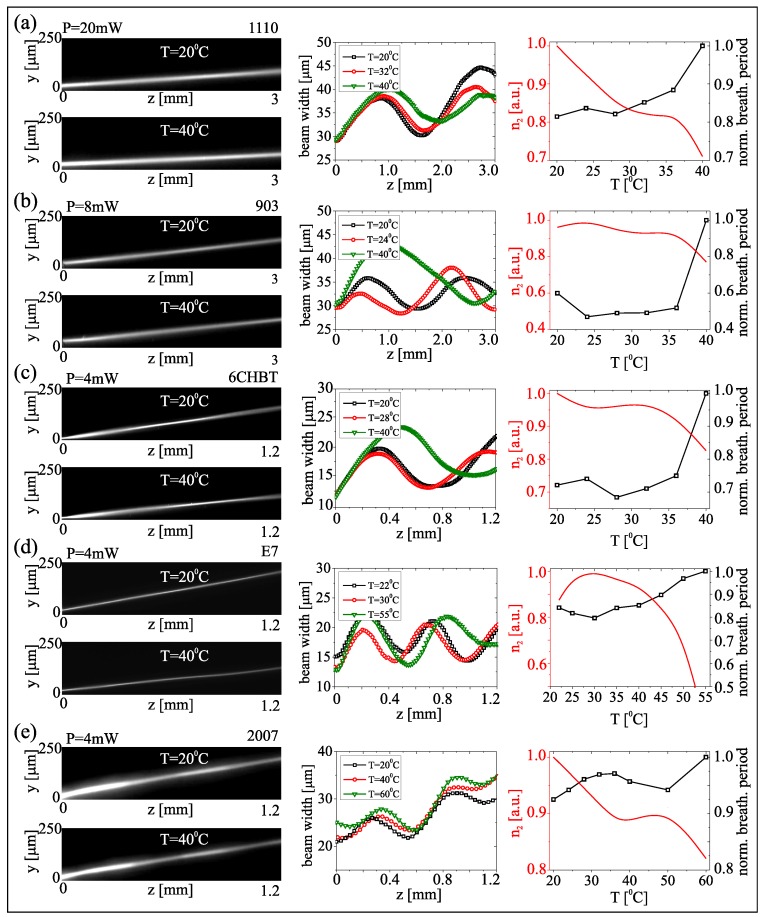
(**Left**): acquired images of beam evolution in the plane yz at two temperatures in each mixture from (**a**–**e**), respectively, as labeled. (**Center**): measured beam width versus propagation at various temperatures (see legends). (**Right**): calculated parameter $n_{2}$ and measured breathing period after scaling to the maximum value.

Figure 4 links input powers and propagating features (trajectories, widths vs. z) of the beams in the five mixtures, together with graphs (Figure 4b) of the calculated equivalent nonlinearity introduced in Equation (6), which cannot be directly extracted from the acquired beam evolution. Conversely, the beam width (FWHM) is an observable linked to confinement and thus to the nonlinearity. Since nematicons tend to breathe, width and amplitude oscillations of their profile relate to the nonlinear response, the latter inversely proportional to the periodicity [51,52]: Figure 4 shows that, indeed, the higher the nonlinear parameter, the shorter the breathing period. When changing the opto-mechanical properties of the material via temperature, the reorientation dependence on power changes, as well. Figure 5 presents the calculated nonlinearity and the measured breathing period versus temperature. Since elastic and dielectric constants and their temperature dependencies differ in each mixture, the trend of nonlinearity with temperature is non-trivial but the expected inverse relationship is verified.

**Figure 6:**
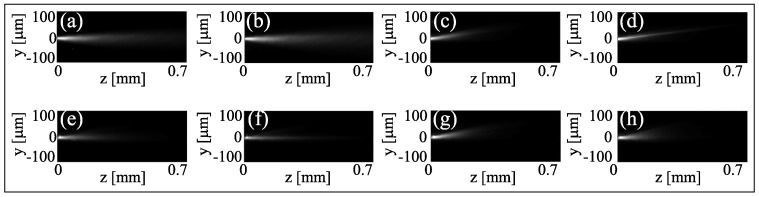
Acquired images of beam evolution at two wavelengths: top, $\lambda_{i}=1064$ nm, bottom, $\lambda_{g}=532$ nm. (**a**,**b**) The near-infrared beam diffracts when polarized as an ordinary wave, irrespective of the power; (**c**,**d**) when extraordinarily polarized, the beam goes from diffraction at low power (P<1 mW) in (**c**) to self-confinement for P=4 mW in (**d**). (**e**,**f**) The green beam (within the absorption band of the DDNLC) in the ordinary polarization undergoes self-focusing when increasing power from $P_{g}=1$ mW (**e**) to $P_{g}=6$ mW (**f**); (**g**,**h**) when polarized as an extraordinary wave it self-defocuses.

While the temperature was an external stimulus to tune the response above, the beam-matter interaction can also be exploited to induce thermo-optic effects via absorption, as implied by Equation (7). Since the NLC mixtures employed for the previous analysis exhibit negligible absorption and therefore the thermo-optic nonlinearity could not be accessed in the used power interval, we added a small amount of absorbing dye to 6CHBT (see the Materials and Methods section). Such dye-doped NLC gues-host (DDNLC) exhibited a strong thermal response in the visible spectrum, while still allowing for beam observation during propagation; Figure 6 shows the evolution of two beams at $\lambda_{i}=1064$ and $\lambda_{g}=532$ nm, respectively, with comparable waists of 3 μm in either ordinary- or extraordinary-wave polarizations.

As mentioned earlier, the thermo-optic variations of ordinary and extraordinary indices have opposite signs as compared to reorientation, e.g., the temperature-induced index profile is focusing for an ordinary wave. Figure 6a–d shows the near-infrared beam diffracting when ordinary polarized (a–b) or at (low) powers P<1 mW and self-confined when extraordinarily polarized for P=4 mW (c–d). Conversely, the green beam undergoes self-focusing when power is raised in the ordinary polarization (Figure 6e,f), self-defocusing as an extraordinary wave (Figure 6g,h) compared to the linear limit (P<<1 mW). In the extraordinary-wave polarization, the role of temperature variations on the trajectory is visible as well: the decrease in birefringence lowers the walk-off δ, with an angular displacement (Poynting vector with respect to the wave-vector) gradually reducing by ≈$2^{\circ }$.

**Figure 7:**
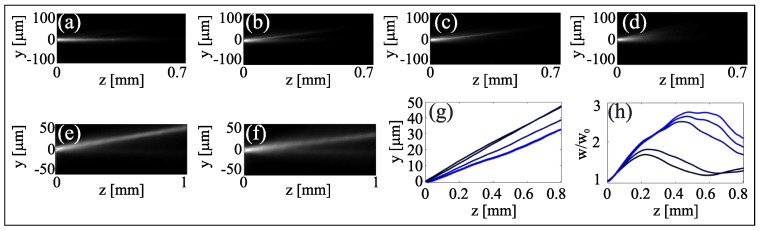
Competing nonlinearities in DDNLC. (**a**–**c**) Acquired images of green beam evolution for various linear input polarizations and $P_{g}=6$ mW, in the presence of a $P_{i}=4$ mW NIR nematicon. (**a**) In the ordinary polarization, the visible beam self-confines and does not sense the extraordinary-wave nematicon waveguide. (**b**) When polarized at $45^{\circ }$ the ordinary component is less confined (owing to reduced power) while the extraordinary component gets confined within the nematicon, as in the case (**c**) of both co-polarized extraordinary-wave beams. (**d**) The $P_{g}=6$ mW extraordinary-wave green without NIR undergoes self-defocusing. (**e**,**f**) Acquired images of the NIR nematicon ($P_{i}=4$ mW) interacting with a collinear co-polarized green beam for (**e**) $P_{g}=1$ mW and (**f**) $P_{g}=6$ mW. NIR beam (**g**) trajectories and (**h**) width evolutions when varying the green power from 0 to 6 mW (darker to lighter lines), respectively.

In DDNLC, thermal and reorientational responses can also operate in synergistic/competing manners when exploiting beam self-action at both resonant and non-resonant wavelengths. A first example is presented in Figure 7a–d: a near-infrared (NIR) extraordinary-wave beam at $\lambda_{i}=1064$ nm is launched in the dye-doped mixture at a power $P_{i}=4$ mW, large enough to generate a nematicon; a second beam at $\lambda_{g}=532$ nm is collinearly injected at $P_{g}=6$ mW. When the latter is ordinarily polarized, it does not sense the refractive index profile generated by the NIR and the thermal response triggers self-focusing: the two nonlinearities are decoupled. Rotating the linear input polarization of the green beam, the power in the ordinary wave decreases and the beam starts spreading, while its extraordinary-wave component gets confined within the NIR nematicon waveguide. The interaction between the two nonlinearities manifests on the trajectory: the walk-off changes when both beams are extraordinary waves, as the temperature is slightly raised by the green component. Moreover, from the confinement of the extraordinary-wave green beam we can infer that reorientation (focusing) dominates over the thermal (defocusing) response.

We further investigated the two competing nonlinear effects by co-launching two extraordinarily-polarized beams at 532 and 1064 nm, respectively, as shown in Figure 7e–h. In this case we slightly tilted the green beam in the plane yz, in order to match the different walk-off at the NIR and so maximize the spatial overlap of the two inputs. The power of the NIR beam was set to $P_{i}=4$ mW, the power of the green varied between 0 and 6.5 mW. For $P_{g}=0$ mW a nematicon was generated by the NIR. Increasing the green power, thus introducing nonlinear defocusing, we observed changes in nematicon propagation as shown in Figure 7e,f. As apparent in Figure 7g, the beam trajectory was steered towards smaller walk-offs, as expected for a thermally driven decrease of birefringence. The resulting measured walk-off angles differed by $\approx 2^{\circ }$, indicating a temperature rise of about $35\;{}^{\circ }$C for $P_{g}=6$ mW. We also measured the breathing period: as the green power increased, the period decreased, Figure 7h, as expected for a defocusing nonlinearity.

These measurements indicate that the two competing nonlinearities (reorientational and thermal) act on different power levels and can be used independently, with the thermo-optic response able to modulate reorientation and thus beam confinement.

## 4. Discussion

We have investigated thermal effects on linear and nonlinear light beam propagation in several nematic liquid crystal mixtures, comparing expected and measured trends of beam walk-off and nonlinear confinement as a function of temperature. We could control the (self-confined) beam trajectory in the near-infrared by simply varying the sample temperature while obtaining the simultaneous modulation of the confinement strength. The excellent agreement between calculated and experimental values in all the NLC mixtures demonstrates the simplicity of such control over reorientational solitons by means of thermo-optic adjustments. In a dye-doped nematic liquid crystal, we exploited the combination/competition of the two nonlinear processes acting on different power scales in order to modulate the propagation properties of a beam: reorientation -stemming from non-resonant light-matter interaction- and absorption -yielding a thermo-optic nonlinearity- were synergistically employed. All the gathered results contribute to a better comprehension of the competition/synergy of nonlinear mechanisms in birefringent soft-matter and their exploitation in controlling beam propagation. These findings are likely to impact on novel generations of signal processors based on light guiding in soft matter, including liquid crystals, polymers, colloidal suspensions, etc.

## 5. Materials and Methods

The planar cells we used for the measurements were prepared with 1.1 mm thick BK7 glass slides; the sample lengths (along *z*) were 1.2 to 3 mm and the thickness -defined by the slide separation across *x*- was 100 μm. The inner interfaces were spun with polymer and mechanically rubbed to ensure a uniform director anchoring (and consequent bulk orientation) at $60^{\circ }$ with respect to *z*. After assembling, the cells were filled with various NLC mixtures by capillarity, avoiding the formation of air bubbles/gaps near the boundaries. An input glass interface ensured molecular anchoring with director along y in z=0 mm and avoided meniscus formation and undesired beam depolarization. In nematicon experiments we used a finite Gussian beam from a Nd:YAG laser operating at λ=1.064 μm and linearly polarized at the input with electric field along y, focused by a microscope objective (20×) in z=0 to a waist $w_{0}\approx 3\;\mu$m in the midplane (x=50 μm between upper and lower glass/NLC interfaces). The beam propagation in the principal plane yz was monitored with a high-resolution CCD camera, imaging the light scattered out of the observation plane. A Gaussian green beam from a frequency-doubled Nd:YAG laser was used in experiments on thermal self-action. Waveplates and linear polarizers were employed to control the relative component polarizations and powers.

The sample temperature was set/stabilized with a proportional-integral differential (Hanyoung MX4) controller enabling a resolution of $0.5\;{}^{\circ }$C. Due to the lack in literature of complete measurement sets for each of the used NLC mixtures, we resorted to an Abbe refractometer to measure the refractive indices. The Cauchy model and Haller’s approximation were use to calculate their wavelength dispersion and temperature dependencies, respectively. The elastic constants and their temperature variations were measured as in Reference [53]. Table 1 presents the measured values of temperature-dependent optical and mechanical parameters of the five mixtures, as graphed also in Figure 1.

In the analysis of competing nonlinearities, we used the 6CHBT mixture as a host material, doped with 0.5% weight of *Sudan Blue II* dye to enhance absorption and thermal effects. Such dye has an absorption peak at λ≈604 nm [54]. The *Sudan Blue II* dye modifies neither the refractive indices nor the elastic constant of the host.

## Figures and Tables

**Table 1:** Measured values of refractive indices and Frank elastic constant K22 for different temperatures.

**1110**				**6CHBT**				**903**			
T [${}^{\circ }$]	$n_{⊥}$	$n_{\left|\right|}$	$K_{22}\left[pN\right]$	T [${}^{\circ }$]	$n_{⊥}$	$n_{\left|\right|}$	$K_{22}\left[pN\right]$	T [${}^{\circ }$]	$n_{⊥}$	$n_{\left|\right|}$	$K_{22}\left[pN\right]$
20	1.4517	1.4976	8.36	20	1.4967	1.6335	3.61	20	1.4696	1.5422	7.36
24	1.4506	1.4947	7.95	24	1.5021	1.6314	3.44	24	1.4681	1.5410	7.17
28	1.4496	1.4916	7.38	28	1.5046	1.6262	3.01	28	1.4662	1.5371	6.95
32	1.4488	1.4882	5.98	32	1.5097	1.6203	2.55	32	1.4649	1.5345	6.68
36	1.4482	1.4844	4.72	36	1.5135	1.6138	2.14	36	1.4626	1.5317	6.35
40	1.4480	1.4797	3.65	40	1.5161	1.5923	1.64	40	1.4609	1.5269	5.96
**2007A**				**E7**							
T [${}^{\circ }$]	$n_{⊥}$	$n_{\left|\right|}$	$K_{22}\left[pN\right]$	T [${}^{\circ }$]	$n_{⊥}$	$n_{\left|\right|}$	$K_{22}\left[pN\right]$				
20	1.5090	1.7773	15.01	20	1.5290	1.7314	4.39				
24	1.5091	1.7739	14.79	25	1.5340	1.7298	3.70				
28	1.5093	1.7704	14.50	30	1.5393	1.7276	3.33				
32	1.5096	1.7668	14.16	35	1.5450	1.7246	3.05				
36	1.5100	1.7630	13.78	40	1.5513	1.7203	2.85				
40	1.5105	1.7590	13.37	45	1.5586	1.7141	2.59				
50	1.5122	1.7580	12.25	50	1.5679	1.7039	2.33				
60	1.5149	1.7450	10.85	55	1.5856	1.6770	2.22				
